# Minimal Influence of [NiFe] Hydrogenase on Hydrogen Isotope Fractionation in H_2_-Oxidizing *Cupriavidus necator*

**DOI:** 10.3389/fmicb.2017.01886

**Published:** 2017-10-04

**Authors:** Brian J. Campbell, Alex L. Sessions, Daniel N. Fox, Blair G. Paul, Qianhui Qin, Matthias Y. Kellermann, David L. Valentine

**Affiliations:** ^1^Department of Earth Science, University of California, Santa Barbara, Santa Barbara, CA, United States; ^2^Division of Geological and Planetary Sciences, California Institute of Technology, Pasadena, CA, United States; ^3^Undergraduate College of Letters and Sciences, University of California, Santa Barbara, Santa Barbara, CA, United States; ^4^Marine Science Institute, University of California, Santa Barbara, Santa Barbara, CA, United States; ^5^Interdepartmental Graduate Program in Marine Science, University of California, Santa Barbara, Santa Barbara, CA, United States; ^6^Institute for Chemistry and Biology of the Marine Environment, Carl von Ossietzky University, Wilhelmshaven, Germany

**Keywords:** *Cupriavidus necator*, hydrogenase, hydrogen isotope, D/H, fatty acid, autotrophic metabolism

## Abstract

Fatty acids produced by H_2_-metabolizing bacteria are sometimes observed to be more D-depleted than those of photoautotrophic organisms, a trait that has been suggested as diagnostic for chemoautotrophic bacteria. The biochemical reasons for such a depletion are not known, but are often assumed to involve the strong D-depletion of H_2_. Here, we cultivated the bacterium *Cupriavidus necator* H16 (formerly *Ralstonia eutropha* H16) under aerobic, H_2_-consuming, chemoautotrophic conditions and measured the isotopic compositions of its fatty acids. In parallel with the wild type, two mutants of this strain, each lacking one of two key hydrogenase enzymes, were also grown and measured. In all three strains, fractionations between fatty acids and water ranged from -173‰ to -235‰, and averaged -217‰, -196‰, and -226‰, respectively, for the wild type, SH^-^ mutant, and MBH^-^ mutant. There was a modest increase in δD as a result of loss of the soluble hydrogenase enzyme. Fractionation curves for all three strains were constructed by growing parallel cultures in waters with δD_water_ values of approximately -25‰, 520‰, and 1100‰. These curves indicate that at least 90% of the hydrogen in fatty acids is derived from water, not H_2_. Published details of the biochemistry of the soluble and membrane-bound hydrogenases confirm that these enzymes transfer electrons rather than intact hydride (H^-^) ions, providing no direct mechanism to connect the isotopic composition of H_2_ to that of lipids. Multiple lines of evidence thus agree that in this organism, and presumably others like it, environmental H_2_ plays little or no direct role in controlling lipid δD values. The observed fractionations must instead result from isotope effects in the reduction of NAD(P)H by reductases with flavin prosthetic groups, which transfer two electrons and acquire H^+^ (or D^+^) from solution. Parallels to NADPH reduction in photosynthesis may explain why D/H fractionations in *C. necator* are nearly identical to those in many photoautotrophic algae and bacteria. We conclude that strong D-depletion is not a diagnostic feature of chemoautotrophy.

## Introduction

The stable hydrogen isotope (D/H) ratios of *n*-alkyl lipids vary widely among the organisms that produce them. In plants and algae, fractionation between water and lipid hydrogen generally falls in a range of roughly –130‰ to –260‰ (Sessions et al., 1999; Chikaraishi et al., 2004; Zhang and Sachs, 2007; Chivall et al., 2014; Heinzelmann et al., 2015a). In heterotrophic microbes, the fractionation is typically smaller, and sometimes even reversed, leading to characteristically D-enriched lipids. Zhang et al. (2009a) further suggested that organisms using TCA-cycle substrates would produce more D-enriched lipids than those using glycolytic substrates, and this pattern has been largely confirmed in a number of other species of aerobic heterotrophs (Dirghangi and Pagani, 2013; Dawson et al., 2015; Heinzelmann et al., 2015b; Kopf et al., 2015; Osburn et al., 2016).

In contrast, chemoautotrophs have been studied much less frequently. Valentine et al. (2004) reported on *Sporomusa* sp., an anaerobic H_2_-consuming acetogen that exhibits very large D/H fractionations (>400‰) relative to water. Campbell et al. (2009) grew *Desulfobacterium autotrophicum*, an obligate anaerobe, on H_2_ + CO_2_ or on formate and observed D-depletions of lipids generally between 260 and 390‰. Fatty acids in cultures grown on H_2_ were only slightly (20–30‰) D-depleted relative to those grown on formate. Zhang et al. (2009a) presented data for *Cupriavidus oxalaticus* and *C. necator* grown aerobically on oxalate and formate, both of which lead to fractionations of 250–300‰. Heinzelmann et al. (2015b) grew *Thiobacillus denitrificans* on thiosulfate and measured fatty acids depleted in D by 217–275‰ relative to growth water. Osburn et al. (2011) presented data from a series of hot springs in Yellowstone National Park, in which C_20_ fatty acids were positively attributed to members of the order Aquificales. In this setting, Aquificales organisms are thought to grow as aerobic hydrogenotrophs, while their fatty acids exhibit D-depletions relative to ambient water of 250–300‰.

Together these findings suggested a pattern in which chemoautotrophs are D-depleted relative to photoautotrophs, but by quite variable amounts. Larger depletions seemed to be generally associated with anaerobes and/or with hydrogenotrophy, although no mechanistic understanding of those patterns has yet been proposed. H_2_ is very strongly D-depleted relative to water at equilibrium, by >600‰ (Horibe and Craig, 1995), providing a possible source for lower D/H ratios in hydrogenotrophs. Yet in the study of *D. autotrophicum*, Campbell et al. (2009) found that H_2_ contributed no hydrogen to lipids. Conversely, in cultures grown in D_2_O, the hydrogenotrophic methanogen *Methanothermobacter thermautotrophicus* derives part of its methane hydrogen indirectly from H_2_ via the hydrogenase-catalyzed release of H^+^ into the intracellular aqueous proton pool (Spencer et al., 1980). It thus remains unclear whether – or why – hydrogenotrophy should be associated with D-depletion of lipids.

To study such issues, we cultivated three strains of *C. necator* aerobically on H_2_ + CO_2_: strain H16 wild type, a mutant lacking the membrane-bound hydrogenase (MBH^-^), and a mutant lacking the soluble hydrogenase (SH^-^). Both of these hydrogenases catalyze the reversible, heterolytic oxidation of H_2_ to 2H^+^ + 2*e*^-^ and are involved in energy generation. These mutants allowed a direct test of the involvement of hydrogenase enzymes in generating strongly D-depleted lipid signatures. We monitored the hydrogen isotopic composition of H_2_ during the growth of each culture and found that it shifted toward equilibrium with H_2_O. Parallel cultures grown in waters of varying D/H allowed us to indirectly assess the contributions of H_2_ and H_2_O to lipid hydrogen.

## Materials and Methods

### Bacterial Strains and Experimental Conditions

*Cupriavidus necator* H16 (DSM 428, ATCC 17699) is a Gram-negative, aerobic, facultatively lithoautotrophic, H_2_-oxidizing bacterium that has been widely studied and reported under previous names, including *Hydrogenomonas* H16, *Alcaligenes eutrophus* (Davis et al., 1969), *Ralstonia eutropha* (Yabuuchi et al., 1995), and *Wautersia eutropha* (Vaneechoutte et al., 2004). Most recently, *W. eutropha* was reassigned to the prior taxon *C. necator* (Vandamme and Coenye, 2004).

*C. necator* H16 was obtained from the German Collection of Microorganisms and Cell Cultures (DSMZ), as were two mutant strains isolated by Pfitzner (1974): SH^-^ (strain LH^-^7, DSM 416) and MBH^-^ (strain PH^-^9, DSM 418) (Schink and Schlegel, 1978). All strains were grown in phosphate-buffered mineral media for chemolithotrophic growth (DSMZ Medium 81). In the main experiment, each strain was grown in three separate cultures having no, moderate (∼500‰) and high (∼1100‰) levels of D-enrichment in the medium water. Culture media of differing D/H ratio were prepared by volumetric dilution of a 10% D_2_O stock to the required level (Campbell et al., 2009). To better understand variability, we conducted a second experiment in which *C. necator* H16 and the two hydrogenase mutants were cultivated in triplicate. These triplicate cultures of each strain were prepared without D-enrichment of the medium water.

Culture conditions were chosen to balance the following objectives: (1) maximize biomass available for isotopic analysis, (2) minimize shifts in isotopic composition of water and H_2_, and (3) safely handle combustible mixtures of H_2_ + O_2_. Cultures were therefore grown in moderate-sized batch cultures with headspace replenished once per day, as follows. The 200-mL cultures were grown in ∼1050-mL borosilicate glass bottles modified for closure with butyl rubber stoppers (13-mm inner diameter, 20-mm outer) and aluminum crimps. Before inoculation of sterile media, headspace gas was replaced with H_2_ + CO_2_ (80:20 by volume). Gas was sterilized by flow through a sterile, cotton-packed syringe after removal of trace O_2_ by flow through a heated steel column containing reduced copper filings (Hungate, 1969). The 250 mL of filter-sterilized atmospheric air was added to the headspace via syringe, yielding a slight overpressure and headspace gas composition of approximately 62% H_2,_ 15% CO_2_, 5% O_2_, and 18% N_2_ (by volume). Headspace and liquid media were allowed to equilibrate overnight before inoculation.

Starter cultures of each strain were grown to optical density ≥ 1 (OD, 660 nm) in a single culture with δD_water_ ≈-25‰. To minimize the contribution of these cells to final biomass, they were diluted ∼10-fold by transfer into sterile medium (δD_water_ ≈-25‰). From this dilute culture, 1 mL was used to inoculate 200 mL sterile medium.

Cultures were incubated at 31°C with shaking at 100 rpm. Headspace gas composition was maintained by daily replacement with H_2_/CO_2_ and addition of air (as described above). OD was measured only once per day to minimize interference with the isotopic composition of headspace gases. The cultures of each strain were harvested when all three cultures exhibited OD > 0.9. Except where noted, collection and storage of samples for D/H analyses followed the protocol of Campbell et al. (2009). Water samples were collected from each culture immediately after inoculation and immediately before harvest. A single gas sample of ∼10 mL was collected from each bottle immediately prior to harvest. In the first experiment, wild-type and MBH^-^ cultures were harvested after collection of final H_2_ and water samples on the seventh day after inoculation. SH^-^ cultures were similarly harvested on the 16th day after inoculation. In the second experiment, all cultures were harvested after collection of final H_2_ and water samples on the 23rd day after inoculation. For the first experiment, biomass was collected via vacuum filtration onto precombusted glass-microfiber filters (Whatman GF/F, 0.7-μm nominal pore size). Filtration apparatus was soaked in 5% HCl and rinsed thoroughly with milli-Q water between harvesting successive cultures. For the second experiment, biomass was pelleted by centrifugation and kept frozen prior to extraction.

### Fatty Acid and Polar Lipid Analyses

Harvested biomass for fatty acid analyses was first lyophilized, then saponified in 1M KOH/H_2_O (70°C, 6 h) to provide the maximum yield of free fatty acids. pH was adjusted to <2 with 6M HCl, and the fatty acids were extracted into methyl t-butyl ether (Valentine et al., 2004). In the main experiment, a 10% aliquot of the extract from each sample was derivatized as the trimethylsilyl (TMS) ethers by reaction with bis-trifluoroacetamide (BSTFA) at 40°C for 15 min, and analyzed by gas chromatography/mass spectrometry (GC/MS) on a Thermo Trace GC/DSQII MS. Fatty acid TMS ethers were identified from their mass spectra and retention times by comparison to library spectra and authentic standards. Double bond position for the 18:1Δ9 fatty acid was confirmed by analysis of the picolinic ester, which fragments distinctively at the double bond position (Christie, 2003). The cyc-17:0, cyc-19:0, and OH-14:0 fatty acids were identified by comparison to an authentic standard of bacterial fatty acids (Sigma Chemical, St. Louis, MO, United States). Fatty acids were quantified as their TMS ethers by splitting ∼30% of the GC effluent to a flame ionization detector (FID). Samples were quantified relative to an internal standard (palmitic acid isobutyl ester) assuming equal response factors for all compounds. In the second experiment involving triplicate cultures in water without D-enrichment, fatty acids were derivatized and quantified as fatty acid methyl esters only. Hydroxyl-fatty acids were not quantified for this experiment.

To provide a more comprehensive understanding of membrane lipid composition, biomass from the second experiment was also subjected to polar lipid analysis. Biomass was first lyophilized and then aliquots were extracted according to a modified Bligh and Dyer protocol (Kellermann et al., 2016). In brief, a combination of lyophilized cell pellets and 3 g of pre-combusted silica sand were extracted in four cycles by ultrasonication using a solvent mixture (ratio 2:1:0.8; v:v:v) of methanol (MeOH), dichloromethane (DCM), and aqueous buffer solution. A phosphate buffer (8.7 g l^-1^ KH_2_PO_4_, pH 7.4) was used for the first two and a trichloroacetic acid buffer (50 gl^-1^ TCA, pH 2) for the last two extraction steps. After each extraction, the solution was separated by centrifugation and all four supernatants were consecutively pooled in a separatory funnel, in which DCM and water had been added to facilitate optimal phase separation. After transfer of the organic phase, the remaining aqueous phase was extracted two more times with DCM. Finally, the pooled organic layers were washed three times with deionized milliQ water and the total lipid extract evaporated under a stream of N_2_ and stored at -20°C.

For polar lipid analysis, an aliquot of the total lipid extract was dissolved in MeOH:DCM, (9:1, v:v) and analyzed using a quadrupole time-of-flight mass spectrometer (Q-ToF-MS). Chromatographic separation was achieved on a Waters Acquity BEH C18 column (1.7 μm, 2.1 × 150 mm) with an ACQUITY Ultra Performance Liquid Chromatography (UPLC) H-Class System (Waters Co., Milford, MA, United States) coupled to a Synapt G2-Si HDMS high-resolution Q-ToF-MS (Waters Co., Manchester, United Kingdom) equipped with a LockSpray dual electrospray ion source operated in both positive (POS) and negative (NEG) ionization modes. The Q-ToF-MS was calibrated in resolution mode over a mass-to-charge (m/z) ranging from 50 to 2000 by using a 0.5 mmol L^-1^ sodium formate solution. For each run leucine enkephalin was used as lock mass, generating a reference ion for POS ([M+H]^+^ = 556.2771) and NEG ionization mode ([M-H]^-^ = 554.2615) to ensure a mass tolerance for all MS or MS/MS experiments of less than 1 ppm. Mass spectral data were collected using the MS^e^ data acquisition function to simultaneously obtain information on the intact molecule (no collision energy applied) as well as their fragmentation data (collision energy ramp reaching from 15 to 75 eV).

Analytes were eluted at a flow rate of 0.4 ml min^-1^ using a linear gradient of MeOH:H_2_O (85:15, v:v, eluent A) to MeOH:isopropanol (50:50, v:v, eluent B) both with 0.04% formic acid and 0.1% aqueous NH_3_ (Wörmer et al., 2013). The initial condition was 100% A held for 2 min, followed by a gradient to 15% B in 0.1 min and a gradient to 85% B in 18 min. The column was then washed with 100% B for 8 min and subsequently returned and held for 5 min to the initial conditions to equilibrate the column for the following run. The column temperature was set to 65°C.

Lipid identification was achieved by analyzing the exact masses of possible precursor ions in positive and negative ionization modes, in combination with their characteristic fragmentation patterns and compound identities described in previous studies (i.e., Kellermann et al., 2011; Tavormina et al., 2017). FA species of the individual polar lipids were identified in negative ionization mode. That is, after collisional activation of a polar lipid, the FA species are liberated with a neutral loss of water to yield an [M - H - 18]^-^ ion. Note that the identification based on MS and MS/MS is a tentative characterization of the analytes. For comparison, all molecular ions (and their adducts) were quantified in positive ionization mode. Since response factors of individual lipid molecules were not accounted for, we only compare relative distribution of lipid species among samples.

### Hydrogen Isotopic Analyses

D/H ratios of water were analyzed with a pyrolysis elemental analyzer (ThermoElectron TC/EA) at UCSB as described by Campbell et al. (2009) and are reported as part-per-thousand (permil, or ‰) deviations from the VSMOW international standard in the conventional δD notation. δD values were calibrated by comparison to two NIST Standard Reference Materials (VSMOW, δD = 0.0‰ and GISP, δD = -189.8‰) and three laboratory working standards of intermediate δD values. The root mean square (RMS) error of δD values for all analyzed standards (*n* = 48) was 3.3‰, which we interpret as the minimum uncertainty in measured values for δD_water_.

Fatty acids were further purified from the remaining (90%) lipid extract by separation into four fractions by solid-phase extraction on an amino-propyl stationary phase following the methodology of Sessions (2006). Fatty acids (fraction 4) were then derivatized as methyl esters by reaction with BF_3_/MeOH (60°C for 10 min), and further reacted with acetic anhydride/pyridine (80°C for 20 min) to produce the acetate derivatives of hydroxyl-fatty acids. D/H ratios of fatty acid esters were determined by reference to both internal (coinjected) and external standard *n*-alkanes similar to those described by Campbell et al. (2009). The pooled standard deviation for replicate analyses of the external *n*-alkane standards was 1.4‰, and the RMS error was 3.3‰ (*n* = 30). The pooled standard deviation for measurements of *n*-alkane standards coinjected with samples, was 3.4‰ (*n* = 54). δD values of FAMEs were corrected for the contributions of methyl and acetyl groups as described by Valentine et al. (2004) and Campbell et al. (2009).

Samples of culture headspace gas for analysis of H_2_ isotope ratio were dried by passing over anhydrous CaSO_4_, then stored in glass serum bottles with butyl rubber stoppers until analysis, 197–206 days. Samples were introduced to the IRMS via a ThermoElectron GasBench device, with δD values measured directly on the H_2_ by comparison to standards (Campbell et al., 2009). Mean precision for unknown samples was 3.4‰.

### Isotope Data Analysis

*C. necator* metabolizes two potential sources of hydrogen: water and H_2_. By varying the isotopic compositions of the inputs, and measuring the concomitant changes in isotopic composition of lipids, we can deduce the relative contributions of those two sources to lipid hydrogen. A complication, however, is that uptake/utilization of hydrogen from either H_2_ or H_2_O is likely accompanied by kinetic fractionations of substantial magnitude. It is not possible to uniquely deconvolve both the relative contributions of those two external sources, and the isotopic fractionations associated with each, using the experimental data available here (see discussion in Sessions and Hayes, 2005). Instead, we follow the approach of Zhang et al. (2009a) in constructing ‘fractionation curves,’ as follows.

Isotopic abundance data for lipids and water reported in delta notation by the Isodat software were first converted to isotope ratios using *R*_x_ = (δD_x_ + 1)*R*_std_, where δD_x_ is the measured δD value of sample *x* and *R*_std_ is the isotope ratio of VSMOW: 0.00015576 (Hagemann et al., 1970). For each fatty acid, we then fit the data from parallel cultures that were grown in water of differing δD values to an equation (Sessions and Hayes, 2005) of the form.

$$R_{FA}=X_{water}\alpha_{FA/water}R_{water}+\left(1-X_{water}\right)\alpha_{FA/H2}R_{H2}$$

where *R* is the isotope ratio of fatty acids, growth water, and hydrogen gas, α is the net isotopic fractionation factor associated with utilization of each hydrogen source, and *X*_water_ is the abundance-fraction of fatty acid hydrogen originating from water (as opposed to H_2_). Briefly, a linear regression of *R*_FA_ against *R*_water_ yields a slope equal to *X*_water_α_FA/water_, and an intercept equal to (1 -*X*_water_)α_FA/H2_*R*_H2_. Since *R*_H2_ is known, (1 -*X*_water_)α_FA/H2_ can be computed directly. This leaves three unknowns (*X*_water_, α_FA/water_, α_FA/H2_) and only two constraints. The set of all possible solutions that satisfy the experimental constraints (for a given fatty acid under constant growth conditions) then define a curve in a plot of α_FA/water_ versus α_FA/H2_, which Zhang et al. (2009a) termed a ‘fractionation curve.’ Further inferences about the magnitudes of the individual fractionations can be drawn from this construct, as discussed in Sections “Results” and “Discussion.”

## Results

### Culture Conditions and Growth

Conditions for the nine independent cultures of the first experiment are summarized in **Table 1**. In cultures with low-D water, δD_water_ increased slightly (4–6‰) over time, while the other treatments exhibited no significant change in δD_water_. Culture media with the lowest δD_water_ values were farthest from isotopic equilibrium with the supplied H_2_, and D-enrichment of water in these cultures would be consistent with hydrogenase-catalyzed isotopic exchange between H_2_ and H_2_O (see discussion in Campbell et al., 2009). For the purpose of estimating fractionations, we adopt the average of the two water δD measurements for each culture (reported in **Table 1**).

**Table 1:** Experimental conditions for growth of *C. necator*.

δD_water_ (‰)^a^	δD_H2_ (‰)			OD 660 at harvest
		
	Supplied	Final	Equilibrium^b^	
**Wild type**				
-25 (1)^c^	-180 (1)	-632 (2)	-736	0.922
523 (2)	-180 (1)	-469 (1)	-587	0.938
1106 (10)	-180 (1)	-364 (3)	-430	0.927
**SH^-^**				
-24 (1)	-180 (1)	-591 (2)	-736	0.964
519 (2)	-180 (1)	-451 (1)	-589	0.984
1104 (2)^∗^	-180 (1)	-355 (1)	-430	0.989
**MBH^-^**				
-24 (1)	-180 (1)	-594 (2)	-736	1.030
524 (2)	-180 (1)	NA	-587	1.042
1110 (5)	-180 (1)	-364 (1)	-429	1.025


*^a^Reported δD_*water*_ value is the average of measurements taken at start and end of culture growth. These values differed by <9‰ in all cultures except one (^∗^), where they differed by 17‰. ^b^Predicted δD_*H2*_ at isotopic equilibrium with supplied water, based on the equation of Horibe and Craig (1995). ^c^Values in parentheses are one standard deviation of replicate analyses of the same sample.*

Values of δD_H2_ changed more dramatically between the last replacement of the headspace and the gas sample taken immediately before harvesting (a period of approximately 1 day). In every culture, δD_H2_ changed by several hundred permil in the direction of equilibrium with water (**Table 1**). The rate of isotopic change was also a function of the extent of H_2_/water disequilibrium, consistent with isotopic exchange as the controlling mechanism for changes in δD values of both water and H_2_. Such shifts in δD_H2_ must have occurred following every replacement of headspace gas, albeit probably to lesser extents when there was less biomass present. These significant shifts in δD_H2_ complicate the analysis of isotopic fractionations, because the harvested biomass must integrate isotopic signals from the changing H_2_. Fortunately, as we show below, the contribution of H_2_ to lipid hydrogen is minor; thus, even these large shifts in δD_H2_ introduce only minor noise into values of δD_FA_ (see section “δD Values of Fatty Acids”).

Growth curves for the cultures were constructed from once-daily measurements of OD, which offered limited resolution of the growth phases. The growth curves for all three strains in both rounds of experiment became pseudo-linear after a brief period of apparently exponential growth (**Supplementary Table S1**). This behavior is consistent with growth limited by O_2_ supply, which precludes calculation of meaningful generation times. Nevertheless, growth of the SH^-^ mutant was appreciably slower than for the other two strains, as has been previously observed (Schink and Schlegel, 1978). Given that all strains received the same daily supply of H_2_ + O_2_, this result presumably reflects a lower growth efficiency of the SH^-^ strain, related to its reliance on reverse electron transport for NAD^+^ reduction (discussed below).

**Table 2** summarizes the conditions for the triplicate cultures of the second experiment, in which the three strains grew in medium water without D-enrichment. The results were similar to those of the first experiment, including impaired growth of the SH^-^ strain relative to the others.

**Table 2:** Summary of δD values (mean, one standard deviation) from triplicate cultures grown in media without D-enriched water.

*C. necator* strain:	Wild type	SH^-^	MBH^-^
**Water**			
Initial	-35 (2)	-37.4 (0.7)	-34 (1)
Final	-15 (1)	-18.6 (0.6)	-13 (2)
Mean	–25	–28.0	–24
H_2_			
Supplied^a^	–181 (2)	–181 (2)	–181 (2)
Final	-472 (10)	-449 (13)	-471 (5)
**Fatty acid**			
14:0	-214 (3)	-211.7 (0.2)	-216 (6)
3-OH14:0^b^	NA	NA	NA
16:0	-204 (1)	-192 (4)	-200 (4)
cyc-17:0	-229 (3)	-218 (4)	-226 (4)
18:1 Δ9	-232 (4)^c^	-207 (3)	-224 (6)
cyc-19:0	-230 (6)	-209 (10)	-219 (9)
Weighted mean	–217	–205	–213


*^a^Duplicate analyses of two separate samples of the gas supplied to all cultures. ^b^Not analyzed (see text); included for continuity with **Table 3**. ^c^Mass sufficient for isotope analysis in two of the three cultures (*n* = 2).*

### Relative Abundances of Fatty Acids and Polar Lipids

**FIGURE 1:**
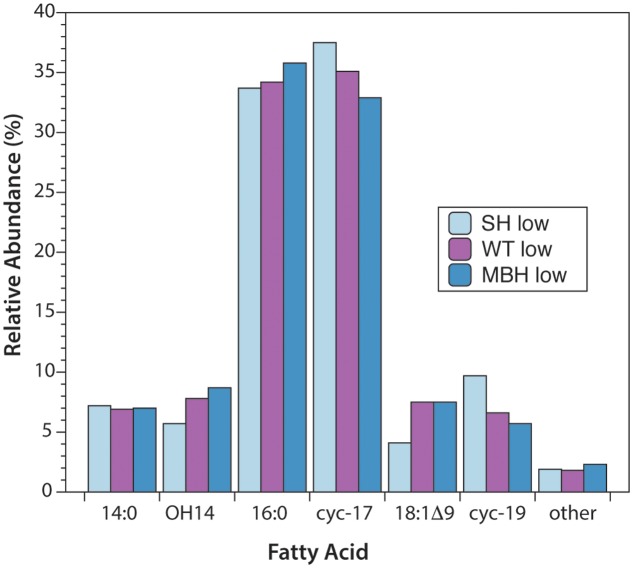
Relative abundances of fatty acids from the three strains of *C. necator*, all grown in non-D-enriched medium. Colors represent the SH^-^ (light blue), wild-type (purple), and MBH^-^ (dark blue) strains.

The predominant lipids in *C. necator* were *n*-alkyl fatty acids. In every strain and culture condition of the first experiment, the same six fatty acids accounted for 98% of the total abundance: tetradecanoic (14:0), 3-hydroxytetradecanoic (OH-14:0), hexadecanoic (16:0), 9,10-methylenehexadecanoic (cyc-17:0), octadec-9-enoic (18:1Δ9), and 9,10-methyleneoctadecanoic (cyc-19:0) acids (**Figure 1**). 16:0 and cyc-17:0 were the most abundant, accounting for ∼70% of total fatty acids. Variations in fatty acid abundance between culture conditions were insignificant, and between strains were minor. The biggest differences between strains were for 18:1Δ9 (3.5% in SH^-^, 7.0% in the wild type, 7.6% in MBH^-^) and for cyc-19:0 (9.3% in SH^-^, 6.5% in the wild type, 5.5% in MBH^-^).

The second experiment yielded the same abundant fatty acids, with the exception that OH-14:0, was not quantified (see section “Fatty Acid and Polar Lipid Analyses”). Relative abundances of the other five predominant fatty acids were comparable to those of the main experiment after exclusion of OH-14:0 from the total abundance. In the second experiment, on average across strains, 16:0 represented approximately 5% more, and 18:1 approximately 5% less, of the total abundance of the predominant fatty acids (excluding OH-14:0). The results were otherwise similar, with the average results for 14:0, cyc-17:0, and cyc-19:0 differing by less than 1% between experiments. Differences between strains were even smaller in the second experiment than in the first. These small differences in the relative abundance of fatty acids are unlikely to affect their H-isotopic compositions. Complete abundance data are provided in the **Supplementary Table S1**.

**FIGURE 2:**
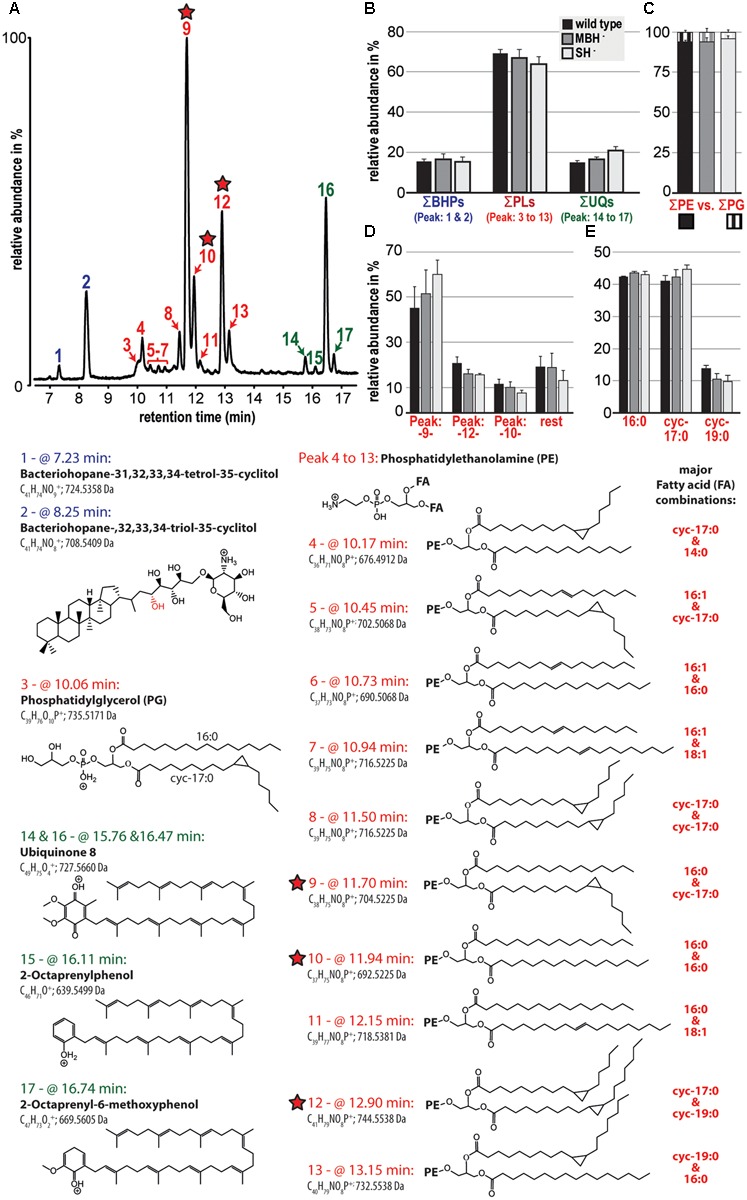
Relative abundances of the polar lipid composition from the three strains of *C. necator* are shown together with their tentative molecular structures. All strains were grown in triplicate and variations among triplicate indicated as standard deviation (black bars in **B** to **E**). **(A)** A partial chromatogram of all identified polar lipid species and **(B)** groups the relative contribution of total bacteriohopanols (BHPs), total phospholipids (PLs), and total ubiquinones (UQs). **(C)** Highlights the dominance of PE-containing headgroups among the total phospholipid fraction and **(D)** shows in more detail the relative contribution of the three most prominent phospholipids (also highlighted by red stars). **(E)** Represents a liquid chromatography-based calculation of the phospholipid-derived fatty acid composition. The applied identifications are based solely on MS and MS/MS and are therefore tentative. Because response factors of individual lipid molecules were not measured, this study compares the relative distribution of lipid species among samples.

The polar lipid composition among the analyzed *C. necator* strains (wild type, MBH^-^, and SH^-^) showed little variation. In all samples, the phospholipids were dominant and comprised >60% of the polar lipid composition, followed by the electron and proton carrier ubiquinone (UQ) and two bacteriohopanols (BHPs; **Figures 2A,B**). The detected phospholipid headgroups were composed of only phosphatidylethanolamine (PE) and phosphatidylglycerol (PG; **Figure 2C**). However, PE clearly dominated at roughly 95% of the phospholipid fraction, with highest relative contributions containing the fatty acid combination of 16:0 and cyc-17:0, 16:0 and 16:0, and cyc-17:0 and cyc-19:0 (**Figure 2D**). Furthermore, the results of this liquid chromatography (LC)-based fatty acid analysis resemble the relative proportions of fatty acids, except for OH-14:0, demonstrated by the GC-based analyses described above. LC-based results also indicate the predominance of 16:0 and cyc-17:0 fatty acids, which together represented greater than 80% of the total (**Figure 2E**).

Polar lipid analysis of biomass from the second experiment indicated that the most significant variation among the *C. necator* strains was an increase in the relative proportion of UQ in the MBH^-^ mutant and foremost in the SH^-^ mutant (**Figure 2B**). The contribution of cyc-17:0 also increased, and that of cyc-19:0 decreased, in the same order among the strains (**Figure 2E**). Complete polar lipid data are provided in the **Supplementary Table S1**.

### δD Values of Fatty Acids

**FIGURE 3:**
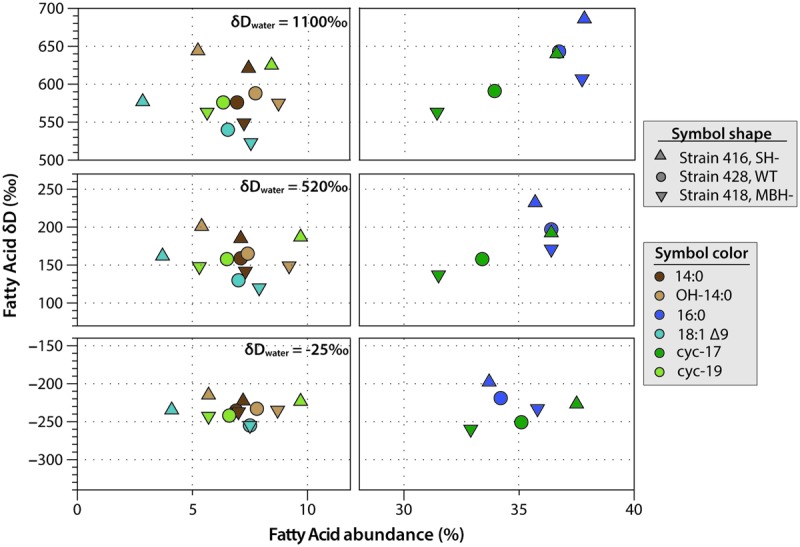
Relative abundance and δD_FA_ values of the six major fatty acids from *C. necator* cultures. The δD_water_ values of culture medium are shown for each group. Error bars (±1 SD) are approximately the size of each symbol, or smaller.

*C. necator* δD_FA_ values ranged from -260‰ to 686‰ (**Figure 3**, **Tables 2**, **3**, and **Supplementary Figure S1**). The range for a given δD_water_ treatment was far narrower, but increased with the δD of the water; i.e., δD_FA_ values were more variable between D-enriched cultures. Analyses of triplicate cultures (second experiment) indicated that biological variability in these δD_FA_ values was generally <5‰, and always <10‰ (1σ). The pooled standard deviation of all fatty acids in all replicate cultures was 5.1‰, a value that we take as representative uncertainty in the following discussion. This is only slightly greater than analytical precision (typically <4‰). In each strain and each culture condition of both experiments, fatty acids were strongly D-depleted relative to growth water, by 178–278‰, with the magnitude of this depletion increasing with δD_water_. In the first experiment (**Table 3** and **Supplementary Table S1**), the abundance-weighted mean fractionation between fatty acids and growth water was -217‰ in the wild type, -196‰ in SH^-^, and -226‰ in MBH^-^ (all values for the δD_water_ = -25‰ growth condition). In the second experiment at similar growth conditions (**Table 2** and **Supplementary Table S1**), abundance-weighted mean fractionations were all shifted to slightly heavier values: -197‰ in the wild type, -182‰ in SH^-^, and -194‰ in MBH^-^. To test whether the small changes in abundance of 18:1 and cyc-19:0 FA between strains could be responsible for such differences, we recalculated weighted mean δD values excluding those two compounds. Differences between the strains remain unchanged when these fatty acids are excluded (not shown).

**Table 3:** Fatty acid δD values from *C. necator* cultures.

*C. necator* strain:	Wild type	SH^-^	MBH^-^
Fatty acid	
**δD_water_ ≈ -25‰^a^**			
14:0	-235.5 (0.5)^b^	-222.8 (0.9)	-237 (3)
3-OH14:0	-233 (2)	-215 (2)	-235 (3)
16:0	-219 (2)	-197.7 (0.4)	-233 (3)
cyc-17:0	-250.8 (0.5)	-226.7 (0.4)	-260 (1)
18:1 Δ9	-255 (1)	-234.6 (0.6)	-255 (2)
cyc-19:0	-242 (4)	-223.2 (0.6)	-243 (6)
Weighted mean^c^	-237	-216	-245
**δD_water_ ≈ 520‰**			
14:0	159 (3)	185 (1)	142 (1)
3-OH14:0	165.1 (0.8)	201 (1)	149 (3)
16:0	197.0 (0.8)	232.3 (0.7)	171 (5)
cyc-17:0	157.9 (0.7)	192.5 (0.8)	137 (3)
18:1 Δ9	130.0 (0.9)	162 (1)	120 (9)
cyc-19:0	157.9 (0.7)	187 (2)	148 (6)
Weighted mean	172	205	150
**δD_water_ ≈ 1100‰**			
14:0	576	621 (3)	549 (5)
3-OH14:0	588	644 (9)	575 (1)
16:0	643	686 (2)	607 (3)
cyc-17:0	591	640 (2)	563 (2)
18:1 Δ9	540	577 (4)	523 (1)
cyc-19:0	576	625 (2)	563 (1)
Weighted mean	605	653	576


*^a^See **Table 1** for precise δD_*water*_ values of each culture. ^b^Values in parentheses are one standard deviation of replicate analyses of the same sample; missing value indicates a single analysis of that sample. ^c^Mean weighted by relative abundance of each fatty acid.*

Comparing δD_FA_ values within each culture, the 16:0 fatty acid was consistently the most D-enriched fatty acid, often by 20–30‰ relative to other fatty acids. In eight of the nine cultures in the main experiment (**Table 3**), OH-14:0 was the second most D-enriched, and 18:1Δ9 was the most D-depleted. For all fatty acids, δD values varied systematically among strains in the order MBH^-^ < wild type < SH^-^, with the three strains typically spanning a range of 30–40‰. That range was broader for cultures grown in D-enriched medium.

The results of the second experiment (**Table 2**) confirmed the relative D-enrichment of the 16:0 fatty acid, but OH-14:0 was not analyzed. These data also confirmed that fatty acids from the SH^-^ strain were significantly more D-enriched than those from the other strains (*p* < 0.002), but did not yield consistent variation in δD_FA_ between the MBH^-^ mutant and the wild type. Accordingly, the differences between wild type and MBH^-^ strains for the δD_water_ = -25‰ experiments are not statistically significant (*p* < 0.19). However, the second experiment included only cultures grown in low-D medium, whereas in the first experiment the difference in δD_FA_ between the MBH^-^ mutant and the wild type was stronger at higher levels of D-enrichment (see **Supplementary Figure S1**). Assuming the biological variance in high-D conditions is similar to that in the low-D experiment, i.e., that σ_δD_ = 5.1‰, the differences between MBH^-^ and the wild type at medium- and high-D enrichments would be significant at *p* < 0.001. Therefore, we retain for discussion the observed systematic variation in δD_FA_ in the order MBH^-^ < wild type < SH^-^.

**FIGURE 4:**
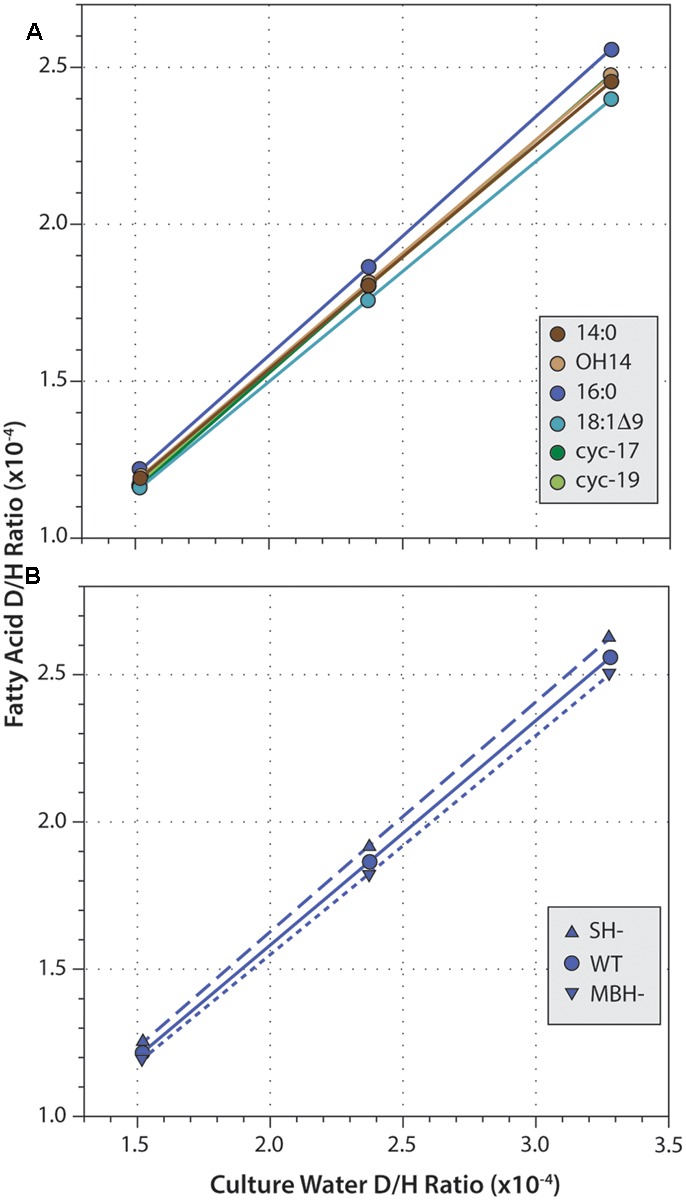
Linear regressions of fatty acid D/H ratio versus growth medium D/H. **(A)** Data for six fatty acids from the wild-type strain. **(B)** Data for 16:0 fatty acid from three strains. Regressions for other data (not shown) are all highly similar.

Fatty acid δD values were strongly correlated with those of water (**Figure 4**; *R*^2^ ≥ 0.9999 and *p* < 0.003 in every regression). Slopes for these correlations ranged from 0.686 to 0.783, consistent with water providing the majority of hydrogen in fatty acids. For all three strains, slopes were highest for 16:0 fatty acid, and lowest for 18:1Δ9 fatty acid. This order follows the pattern of fractionation (16:0 is D-enriched and so less fractionated, thus α is larger; 18:1 is D-depleted and so more fractionated, α is smaller), as would be expected if the proportion of water-derived hydrogen (*X*_water_) remained roughly constant across fatty acids. Similarly, for any given fatty acid, slopes were slightly higher in SH^-^ and slightly lower in MBH^-^ relative to the wild type, again following the pattern of fractionation (SH^-^ is D-enriched and less fractionated; MBH^-^ is D-depleted and more fractionated).

**FIGURE 5:**
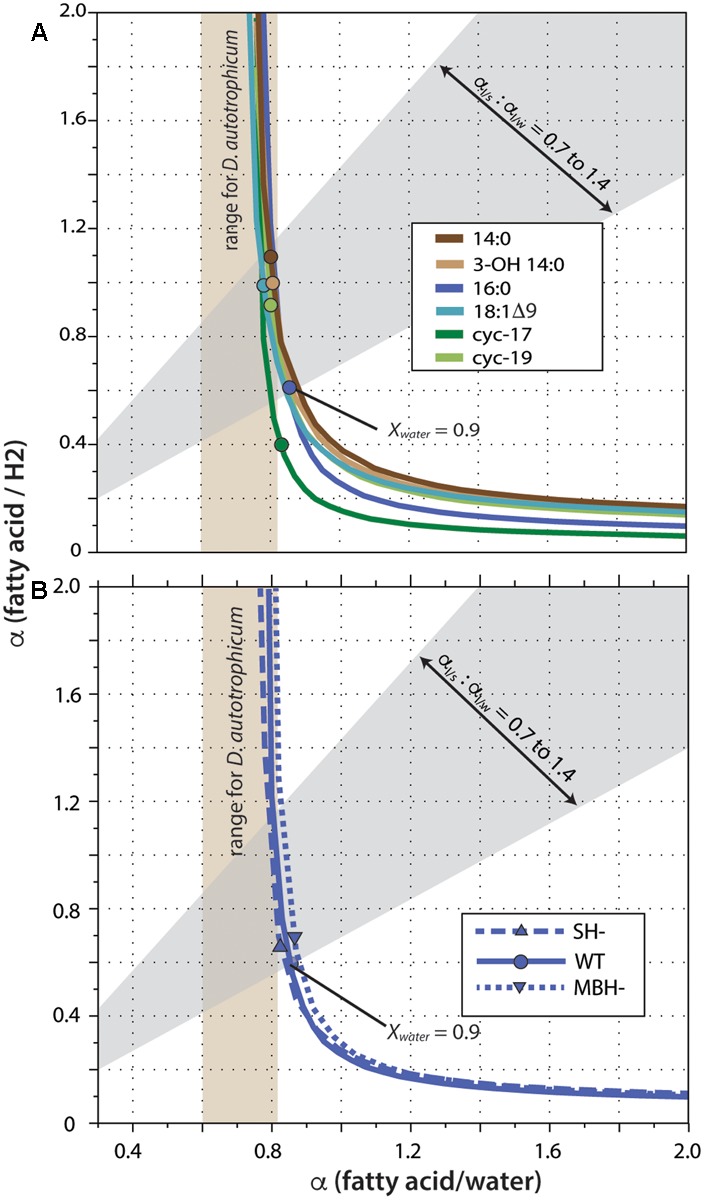
Fractionation curves for different fatty acids from the wild-type strain **(A)**, or for 16:0 fatty acid from different strains **(B)**. Each curve represents the set of all possible combinations of lipid/H_2_ fractionation, lipid/water fractionation, and *X*_water_ (fraction of lipid hydrogen derived from water) that fit a given set of experimental constraints. Circles mark the point on each curve corresponding to the value *X*_water_ = 0.90; these are marked only to facilitate comparison between curves, and do not otherwise have particular meaning. The gray shaded region outlines values where the two fractionations differ by no more than 300‰, as proposed by Zhang et al. (2009a). Tan shaded region indicates lipid/water fractionations measured for *D. autotrophicum* that uses only water hydrogen (Campbell et al., 2009).

Fractionation curves derived from these linear regressions (**Figure 5**) are virtually identical for the same fatty acid from different strains, including the hydrogenase mutants. In contrast, curves for different fatty acids from the same strain are shifted along a nearly vertical trajectory. Such shifts are representative of primarily a change in net lipid/H_2_ fractionation (see discussion in Zhang et al., 2009a), although differences between fatty acids are presumably a manifestation of differences in biosynthetic fractionation rather than differences in metabolic reactions of H_2_ *per se*. Zhang et al. (2009a) suggested that for heterotrophs the two fractionations (lipid/water and lipid/substrate) should likely be within ∼300‰ of each other, a region defined by the gray shading in **Figure 5**. In *C. necator*, the fractionation of H_2_ may be much more significant than that of water, leading to even lower α_FA/H2_ to α_FA/water_ ratios (above and to the left of the gray region). In either case, this analysis indicates that 90% or more of the fatty acid hydrogen was derived from water, not H_2_. The net fractionation between fatty acids and water can also be compared to those of *D. autotrophicum* grown chemoautotrophically; it derives lipid hydrogen exclusively from water, which allowed Campbell et al. (2009) to measure α_FA/water_ directly (tan shaded zone in **Figure 5**). The fractionations for *C. necator* are near the upper end of this range.

A significant complication for this analysis regards the fact that the δD values of headspace H_2_ changed throughout the growth cultures, in a convoluted fashion (because of daily H_2_ additions and continuous equilibration with H_2_O). The curves in **Figure 5** were generated assuming δD_H2_ = –400‰ for all cultures, a value that is at the low end of the averages observed for any single culture (averages ranged from -267 to -406‰; the generation of fractionation curves implicitly assumes a constant value of *R*_H2_ for all cultures). Regardless, the shapes and relative positions of those curves do not depend on the assumed value. The assumed value for δD_H2_ does affect the absolute vertical position (i.e., the implied lipid/H_2_ fractionation factor) of the curves, with a less negative δD_H2_ value moving the curves downward. But since all fatty acids derive from the same H_2_ source, all of the curves move up and down together as δD_H2_ is changed. For our analysis here, the most conservative assumption would be δD_H2_ = –632‰, i.e., the most negative value measured at any time point in any one of the cultures. Under that assumption, the upper bound of the gray shaded region in **Figure 5** would intersect the fractionation curve at *X*_water_ = 0.92, instead of the *X*_water_ = 0.95 obtained by assuming δD_H2_ = –400‰. Assuming higher (less negative) δD_H2_ values would only result in even higher inferred *X*_water_ values. The conclusion that little to no lipid hydrogen derived from H_2_ is thus robust, regardless of the actual, growth-integrated δD value of headspace H_2_.

## Discussion

A first-order observation for *C. necator* grown on H_2_ + O_2_ is that net D/H fractionations of its fatty acids relative to growth medium water fall in the range of -199‰ to -236‰, indistinguishable from those of many photoautotrophic algae and bacteria (Sessions et al., 1999; Chikaraishi et al., 2004; Zhang and Sachs, 2007; Zhang et al., 2009b; Chivall et al., 2014; Heinzelmann et al., 2015a). Deleting either the membrane-bound hydrogenase (MBH) enzyme or the soluble hydrogenase (SH) enzyme has only a small effect on δD_FA_ values, changing them by 25‰ or less (**Figure 3** and **Table 3**). Fractionation curves, constrained by experiments in which the δD of growth water was varied systematically, are also consistent with very little lipid hydrogen (approximately 10% or less) being derived from H_2_, and with lipid/water fractionations in the neighborhood of 200‰ (**Figure 5**). All lines of experimental evidence therefore point to a minimal contribution of H_2_ metabolism to lipid δD values in *C. necator*.

There are, nevertheless, subtle but systematic differences in δD_FA_ values between the wild type and hydrogenase mutants. In our main experiment, fatty acids from SH^-^ were 19.2‰ more D-enriched than those from the wild type (range 18–24‰) in experiments with δD_water_ = -25‰. Differences between cultures grown on more D-enriched water were in the same directions, but even larger (up to 46‰ in magnitude). In comparing fatty acids from MBH^-^ and wild-type strains, there was no significant difference in cultures with δD_water_ = -25‰, but significant differences in more D-enriched cultures. MBH^-^ fatty acids were D-depleted relative to the wild type in those experiments. Such differences cannot plausibly be attributed to changes in fatty acid abundance, as there were barely detectable variations in their relative abundance between the strains. Schouten et al. (2006) and Leavitt et al. (2016) have previously suggested growth rate as an important influence on fractionation, with faster growing strains exhibiting larger fractionations (more negative ε_FA/water_ values). Although growth rates could not be quantified in our experiments (see section “Culture Conditions and Growth”), the relationship between growth rates and fractionations was qualitatively similar to that observed by Schouten et al. (2006) and Leavitt et al. (2016). Notably, however, the size of the effect in the latter study was ∼100‰, whereas in our data it is only 25‰ or less. Somewhat contradictorily, Osburn et al. (2016) have shown for a large survey of anaerobic heterotrophs that, in general, fractionation is not correlated with growth rate, although there is possibly a weak correlation amongst the slowest-growing organisms. The influence of growth rate on D/H fractionations in *Cupriavidus* thus remains uncertain.

Below, we address three remaining questions. First, what role (if any) do hydrogenase enzymes play in setting the isotopic composition of lipids, either in this organism or other hydrogenotrophs? Second, are strong D-depletions truly a hallmark of chemoautotrophic metabolism, as has been previously proposed (Zhang et al., 2009a)? Third, is it coincidence that the δD_FA_ values of *C. necator* are indistinguishable from those of plants?

### Hydrogenase Influence on Fatty Acid Isotopic Compositions

In prokaryotes, including *C. necator*, *n*-alkyl fatty acids are synthesized in the cytoplasm from the precursor acetyl-CoA, which is a key intermediate in many pathways (Heath et al., 2002). During the elongation cycle, hydrogen is transferred to fatty acids from NADPH and – if certain alternative enzymes are present – from NADH (Campbell and Cronan, 2001) during two separate reduction steps. The first, catalyzed by the FabG subunit, reduces 3-ketoacyl-ACP to 3-hydroxyacl-ACP. In most studied organisms this subunit is known to use only NADPH as cofactor. However, the genome of *C. necator* H16 contains at least one FabG with 97% amino acid similarity to a FabG from *Cupriavidus taiwanensis* that uses NADH as cofactor (Javidpour et al., 2014). The second reduction, of enoyl-ACP to 3-hydroxyenoyl-ACP, is catalyzed by the FabI subunit, which is well known to use both NADH and NADPH cofactors. Thus, it appears likely that *C. necator* has the capability of using NADH, probably in combination with NADPH, as a hydride donor for fatty acid biosynthesis.

The immediate sources of hydrogen to fatty acid biosynthesis are thus acetyl-CoA, NAD(P)H, and water (for further discussion, see Osburn et al., 2016). The activities of precursor pathways during autotrophic growth are not known, but in *C. necator* cultivated on gluconate, the major source of NADPH and acetyl-CoA is the oxidation of sugars via the pentose phosphate (PP) pathway (Lee et al., 2003). In *C. necator*, carbohydrate precursors (which flow to PP) are produced by the Calvin–Benson–Bassham (CBB) cycle (Bowien and Kusian, 2002), which in turn uses NADH as the reductant. NADH is itself generated largely from H_2_ via the SH (see below).

**FIGURE 6:**
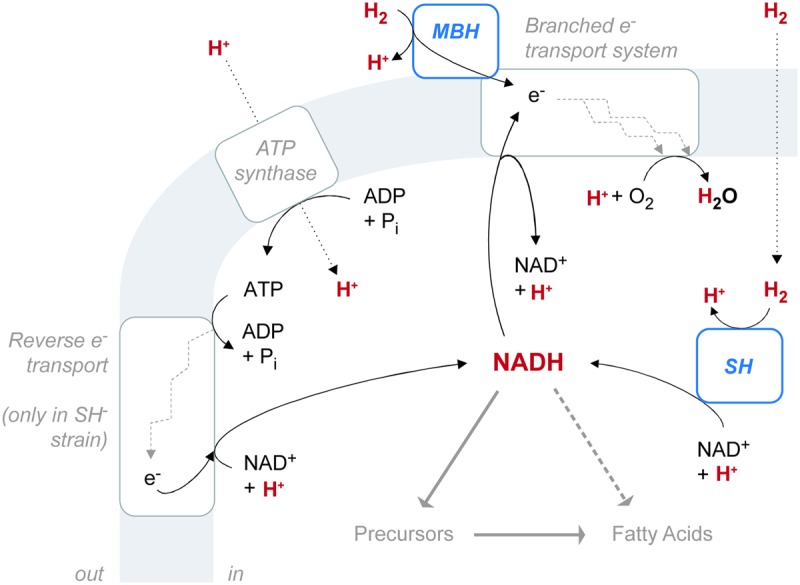
The configuration of hydrogenase enzymes and hydrogen pathways in *C. necator*. The gray shaded area represents the cytoplasmic membrane. ATP synthase and the electron transport system are associated with the membrane, as is the membrane-bound hydrogenase (MBH), which is located in the periplasmic space. The soluble hydrogenase (SH) is located in the cytoplasm. Hydrogen-bearing species are in red. Fine black arrows indicate chemical reactions or half-reactions (not stoichiometrically balanced). Dotted black arrows denote diffusion or transport across the membrane. Heavy gray arrows denote hydrogen flow from NADH to fatty acids, either through biosynthetic precursors (such as NADPH or acetate) or possibly by direct contribution (dashed).

*C. necator* H16 reportedly possesses four putative hydrogenases, each assigned to a different group within the [NiFe] hydrogenase class (Schwartz et al., 2003; Vignais and Colbeau, 2004). The regulatory hydrogenase (RH) belongs to Group 2, the cytoplasmic H_2_ sensors. RH regulates biosynthesis of the two energy-generating hydrogenases and is not directly involved in metabolism (Lenz and Friedrich, 1998). MBH belongs to Group 1, the uptake hydrogenases. The SH belongs to Group 3, the bidirectional, heteromultimeric, cytoplasmic hydrogenases. MBH and SH both generate energy by the heterolytic cleavage of H_2_. In [NiFe] hydrogenases, the process consists of two steps: first H_2_ is cleaved into H^-^ (hydride) and H^+^ ions, and then the two electrons are extracted from the hydride leaving a second H^+^ (de Lacey et al., 2005). The electrons are transferred to an acceptor, while the protons (or deuterons) are transported out of the protein to the surrounding water (Vignais, 2005). Despite these shared properties, MBH and SH differ in both location and function, and they probably have different effects on the pools of water and NADH available for biosynthesis. The reaction network involving hydrogenases and these intermediates must be considered as a whole (**Figure 6**). A fourth [NiFe] hydrogenase (Hyd4) is apparently coded for on the megaplasmid of *C. necator* H16 (Schwartz et al., 2003). However, since the triple mutant lacking RH, MBH, and SH shows no detectable hydrogenase activity, the function of the Hyd4 genes is unknown and is not considered further here.

MBH catalyzes the unidirectional oxidation of H_2_, which is ultimately coupled to O_2_ reduction via a respiratory chain (Schink and Schlegel, 1979). Located in the periplasmic space, MBH is anchored to the cytoplasmic membrane by a *b*-type cytochrome subunit, which is also the immediate acceptor of electrons from H_2_ oxidation (Friedrich and Schwartz, 1993; Bernhard et al., 1997). Electrons from H_2_ flow through the *b*-type cytochrome into the UQ pool, which feeds into the electron transport system. Electron transport complexes link the movement of electrons to the uptake of protons (generating ubiquinol) at the cytoplasmic side of the membrane and the release of protons at the periplasmic side (Komen et al., 1996). The resulting proton gradient supports ATP synthesis.

SH located in the cytoplasm, couples the oxidation of H_2_ to the reduction of NAD^+^ (Schneider and Schlegel, 1976). Reduction is thought to occur via electron transfer through Fe–S clusters of the hoxU subunit, rather than as an intact hydride transfer (Burgdorf et al., 2006). The enzyme is reversible, but H_2_ evolution from NADH, in the presence of sodium dithionite, occurs at only 2.4% of the rate of H_2_ uptake with NAD^+^ as electron acceptor (Schneider and Schlegel, 1976). This property is consistent with the demand for large amounts of NADH by the CBB cycle during autotrophic growth (Bowien and Kusian, 2002). Cells lacking SH can grow autotrophically, but only at a diminished rate, as observed in our study. Impaired growth may be due to inefficient production of NADH via ATP-dependent reverse electron transport (Komen et al., 1992). In our study, the SH^-^ mutant exhibited an increase in relative abundance of UQ over both the wild type and MBH^-^ strains (**Figure 2B**). This observation would be consistent with the upregulation of UQ in SH^-^ cells to stimulate the rate of electron transport in the membrane.

The electron transport system of *C. necator* is central to the relationship of SH, MBH, and the NADH pool. H_2_ oxidation via MBH is not the sole source of electrons for transport. Instead, the system is branched, linking the oxidation of H_2_, NADH, and succinate, through the UQ pool, to four different membrane-bound terminal oxidases (Komen et al., 1991a,b). The two major branches during exponential growth are a *bo*-type oxidase and a cytochrome *c*-containing pathway (which is itself branched, having two terminal oxidases). The fourth terminal oxidase, a *d*-type cytochrome, is active mainly during late exponential growth. In membranes from exponential-phase, autotrophic *C. necator*, electron flow from H_2_ via the *bo*-type oxidase accounts for 80% of ATP synthesis, while the cytochrome *c*-containing pathway plays “a minor role in energy conservation” (Komen et al., 1996). In a study comparing SH^-^ and MBH^-^ mutants (different strains than the mutants in this study) to the wild type, Komen et al. (1992) found that the preferred pathway of electron transport is different for each strain, and the wild type reportedly favors the cytochrome *c*-containing pathway. That study also reported NADH dehydrogenase activity for each strain: MBH^-^ cells exhibited 167% activity relative to wild-type cells, while SH^-^ cells exhibited 30% activity relative to wild-type cells. We speculate that the relative activities of NADH dehydrogenase could be a factor linking growth rates to hydrogen-isotopic fractionations (as shown in Leavitt et al., 2016).

The preceding discussion makes it clear that there is no direct enzymatic link between the isotopic composition of H_2_ and lipids. Both uptake hydrogenases transfer only electrons, not hydrogen nuclei. The fact that SH^-^ mutants are only slightly different in δD_FA_ is consistent with this conclusion. Rather, the modest shifts in δD_FA_ between the wild type, SH^-^, and MBH^-^ are likely due to one or more indirect effects. Several such effects can be considered, including: generation of metabolic water from H_2_, hydrogen-isotopic exchange between water and H_2_, generation of reducing equivalents via reverse electron transport, differing transhydrogenase activity and/or growth rate, and different partitioning of reducing power between catabolism and anabolism.

The different locations of SH and MBH provide two possible mechanisms of influence on the intracellular water pool: first the oxidation of H_2_ to H_2_O, and second the catalyzed isotopic equilibration of H_2_ with H_2_O (i.e., independent of oxidation). MBH, anchored to the outer surface of the cytoplasmic membrane, oxidizes H_2_ in the periplasmic space where the isotopic composition of that signal is presumably quickly mixed with extracellular water and therefore lost. In contrast, SH oxidizes H_2_ in the cytoplasm, where released H^+^ will rapidly equilibrate with water and could thereby be incorporated into fatty acids. We thus can predict that hydrogenase influences on cellular water should be most pronounced in MBH^-^, which relies completely on SH, and should be least pronounced in SH^-^.

Supplied H_2_ in our cultures was always strongly D-depleted relative to growth water, so catabolic oxidation of H_2_ to H_2_O should serve to lower water and thus fatty acid δD values. Assuming that this effect is strongest in the MBH^-^ strain (as argued above), the predicted variation in δD_FA_ would be MBH^-^ < wild type < SH^-^, consistent with the observed variation. Because the isotopic contrast between H_2_ and H_2_O was greatest in those experiments with D-enriched medium, this mechanism could also potentially explain the increasing differences between strains with increasing δD_water._

The second process to consider is hydrogenase-catalyzed isotopic exchange between H_2_ and H_2_O; the existence of this process is clearly indicated by the decrease in δD_H2_ over the course of the experiment. This decrease in δD_H2_ would tend to cause a corresponding enrichment in δD_water_. Indeed, such enrichment was observed directly in the low-δD_water_ treatments, which were farthest from equilibrium with H_2_. Assuming that this effect is strongest in the MBH^-^ strain, the predicted variation in δD_FA_ would be SH^-^ < wild type < MBH^-^. In fact, the opposite order was observed in this study. Therefore, the oxidation of H_2_ to H_2_O is the only mechanism of hydrogenase influence via the intracellular water pool that could yield the observed variation in δD_FA_ between strains. A similar mechanism has previously been observed in *E. coli* (Kreuzer-Martin et al., 2006); however, the metabolic rates that generated a measurable effect in those experiments were likely orders of magnitude faster than those in our experiments. Growth rates in *C. necator*, at least as realized in our O_2_-limited batch cultures, may therefore be too slow to support any significant role for metabolic water.

Another obvious difference between the three *C. necator* strains is the necessity for the SH^-^ mutant to employ reverse electron transport to generate NADH for carbon fixation (**Figure 6**). If this enzymatic reduction expresses a different kinetic isotope effect (KIE) than does SH, then the differing isotopic composition of NADH could be transmitted into carbon fixed by the CBB cycle and ultimately into fatty acids, regardless of whether NADH or NADPH is specifically used by fatty acid synthase. Although this mechanism may contribute partly to the larger difference in δD_FA_ between the SH^-^ mutant and the wild type, it cannot play a role in the difference between the MBH^-^ mutant and the wild type, both of which are thought to reduce NADH via the SH.

The genome of *C. necator* H16 contains at least four putative transhydrogenases, capable of interconverting NADH and NADPH. Given that such enzymes commonly express large KIEs (Jackson et al., 1999), the metabolic balance between NADH and NADPH production has the capacity to significantly affect the δD values of both cofactor pools, and thus fatty acids. We predict that the MBH^-^ mutant would overproduce NADH (because of over-reliance on the NAD^+^-reducing SH), whereas the SH^-^ mutant is demonstrably underproducing NADH. Isotope effects of the specific *C. necator* transhydrogenases have not been reported, but assuming they are similar to most other studied examples, i.e., normal KIEs, overproduction of NADH should emphasize NADH → NADPH and thus D-depletion of NADPH, whereas underproduction should emphasize NADH ← NADPH and D-enrichment of NADPH. If NADPH is the primary cofactor used for fatty acid synthesis, the predicted order of D-enrichment would be MBH^-^ < wild type < SH^-^, which matches the observed data; if NADH were the primary cofactor, the predicted order would be reversed.

Finally, if NADH is used for biosynthesis, it is possible that the competition between anabolic and catabolic demands leads to shifts in isotopic composition. In this model, the NADH pool represents a branch point in the flow of hydrogen from water to lipids. While the hydrogen flux from NADH to lipids is assumed to be similar in all strains (similar FA abundances), there is evidence that the flux from NADH to water (i.e., respiration) varies systematically among strains. This flux represents the oxidation of NADH for electron transport, which is catalyzed by NADH dehydrogenase. As noted above, NADH dehydrogenase activity varies among strains in the order SH^-^ < wild type < MBH^-^ (Komen et al., 1992). If the isotope effects associated with catabolic versus anabolic utilization of NADH differ, which seems plausible, then changes in the branching ratio between these two fates will lead to changes in the isotopic compositions, even with constant NADH supply (see Hayes, 2001). We cannot predict the direction of such effects without knowing the specific isotope effects associated with the two pathways, and either direction is plausible.

In summary, there are several possible mechanisms by which loss of SH or MBH could lead to modest shifts in lipid δD; none of them involve direct transfer of hydrogen isotopes from H_2_ to NAD(P)H. Oxidation of H_2_ to cellular water is possible but, given the slow growth in our experiments, is perhaps unlikely to be significant. Changes in the interconversion of NADH and NADPH, or in the downstream fates of NADH, are plausible. Because hydrogenase enzymes are intimately linked to conservation of cellular reducing equivalents, and thus the NADPH pool, similar linkages are likely in most other organisms that have hydrogenase enzymes.

### Comparison with Other Organisms

**FIGURE 7:**
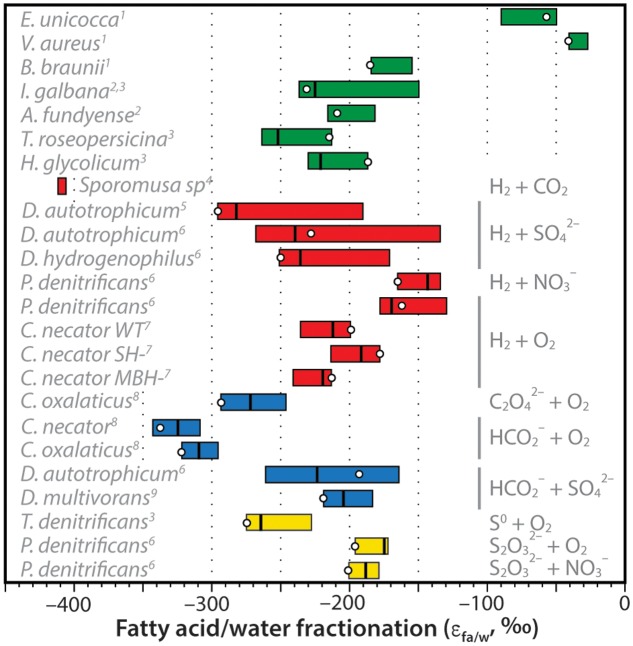
Compilation of measured hydrogen-isotopic fractionations between fatty acids and growth water (expressed as ε_FA/water_ = [(δD_FA_ + 1)/(δD_water_ + 1)]. For each organism, colored bar represents the range of values for all measured fatty acids in that organism, vertical line is the weighted-mean value, circle is the value for palmitic acid (which is often the most abundant FA). Bars are colored coded for photoautotrophs (green), H_2_-using chemoautotrophs (red), formate- or oxalate-using chemoautotrophs (blue), or sulfur-oxidizing chemoautotrophs (yellow). Specific electron donors and acceptors are noted at right, species are noted at left. Data sources: ^1^Zhang and Sachs (2007); ^2^Sessions et al. (1999); ^3^Heinzelmann et al. (2015b); ^4^Valentine et al. (2004); ^5^Campbell et al. (2009); ^6^Osburn et al. (2016); ^7^This study; ^8^Zhang et al. (2009a); ^9^Dawson et al. (2015).

The net D/H fractionations between fatty acids and water exhibited by *C. necator* can be compared to other organisms growing autotrophically (**Figure 7**). These include the marine, unicellular algae *Isochrysis galbana* and *Alexandrium fundyense*; the freshwater green algae *Eudorina unicocca*, *Volvox aureus*, and *Botryococcus braunii*; the photosynthetic purple sulfur bacteria *Thiocapsa roseopersicina* and *Halochromatium glycolicum*; H_2_-consuming cultures of the acetogenic bacterium *Sporomusa* sp. (DMG 58), the sulfate-reducing bacteria *Desulfobacterium autotrophicum* and *Desulfobacter hydrogenophilus*, and nitrate-reducing bacterium *Paracoccus denitrificans*; formate- and oxalate-consuming cultures of *C. necator*, *C. oxalaticus*, *D. autotrophicum*, and *Desulfovibrio multivorans* that are effectively chemoautotrophic with respect to hydrogen sources; and sulfur-oxidizing cultures of the bacteria *Thiobacillus denitrificans* and *P. denitrificans*. Data sources are provided in the caption of **Figure 7**.

Zhang et al. (2009a) had originally hypothesized that substantial D-depletion of lipids relative to growth water was a potential hallmark of chemoautotrophic organisms. This hypothesis was influenced heavily by the early results of Valentine et al. (2004) for *Sporomusa* sp. and Zhang et al. (2009a) for *C. necator* and *C. oxalaticus*, all of which were more D-depleted than photoautotrophs. A precise mechanistic explanation for the D-depletion was not offered. Additional data on autotrophic fractionations (**Figure 7**) are now fairly clear in disproving this hypothesis: D/H fractionations in chemoautotrophs are not significantly different, on average, from those in unicellular photoautotrophs. Fractionations are still larger than those for most terrestrial plants (e.g., Sachse et al., 2012), but this is a consequence of soil and leaf water evaporation rather than a biochemical effect. Some heterotrophs also exhibit lipid/water fractionations that are in a similar range (e.g., Osburn et al., 2016), although many are more D-enriched for reasons that are still not entirely understood.

Although the distributions of chemoautotrophic and photoautotrophic fractionations are largely overlapping, there are several examples of chemoautotrophs that lie well outside the known range for photoautotrophs. These include *D. autotrophicum* grown on H_2_, and *C. necator* and *C. oxalaticus* grown on formate; but most of all, *Sporomusa* emerges as a clear anomaly. With this being the only studied example of a Firmicute or of an acetogenic organism, we do not speculate as to the uniqueness or biochemical basis of its anomalous D-depletion. Regardless, while it is clear that strong D-depletions are not ubiquitous amongst chemoautotrophs, it remains possible that abnormally D-depleted lipids could still be markers for certain metabolic pathways. This clearly requires further study, both to understand the phylogenetic distribution as well as the underlying mechanisms of the signal.

### Similarity of *C. necator* and Algal Fractionations

A conspicuous feature of **Figure 7** is the similarity of fractionations in *C. necator* grown on H_2_ and those in photoautotrophic bacteria and algae. Given that hydrogen metabolism in *C. necator* contributes little or no hydrogen to lipids, this leads us to question whether the similarity is merely a coincidence. Both groups of organisms use the same biosynthetic pathway for fatty acids, fueled by NAD(P)H. However, sources of the latter cofactor differ significantly between knallgas bacteria and photoautotrophs.

In oxygenic photosynthesizers, reducing equivalents are conserved mainly as NADPH during the light reactions of photosynthesis. The electron-transport chain of photosystem I ultimately passes a single low-potential electron to ferredoxin, an iron–sulfur protein. Since iron atoms carry the reducing equivalent, ferredoxin is an electron carrier, not a hydride carrier. Reduced ferredoxin then diffuses to the membrane-bound enzyme ferredoxin-NADP^+^ reductase (FNR). The prosthetic group of FNR is flavin adenine dinucleotide (FAD), which can accumulate two electrons from (two) ferredoxins and two hydrogen nuclei (H^+^ or D^+^) from solution, thereby being reduced to FADH_2_. FADH_2_ will in turn transfer these reducing equivalents as a hydride (H^-^) ion to NADP^+^ with stereospecificity (Ammeraal et al., 1965). The catalytic mechanism of FNR is thought to involve a ternary complex of ferredoxin, FAD, and NADP^+^, and electron/hydride transfer is both rapid and thermodynamically reversible. A large deuterium isotope effect (*k*_H_/*k*_D_ ∼ 6) *in vitro* is consistent with intact hydride transfer (Tejero et al., 2007). The overall direction of the reaction is expected to be pushed strongly in the forward direction under physiologic conditions of photosynthesis, possibly via reductive inhibition by NADPH (Carrillo and Ceccarelli, 2003).

In *C. necator*, reducing equivalents are conserved primarily as NADH, which is generated from NAD^+^ by the SH. SH is a two-domain enzyme, with one module representing the heterodimeric[NiFe] hydrogenase encoded by hoxH and hoxY, and the other a flavin-containing NADH dehydrogenase module encoded by hoxF and hoxU (Burgdorf et al., 2006). The former module accomplishes the oxidation of H_2_, passing electrons (but not hydrogen atoms) to the latter to reduce NAD^+^ to NADH. The NADH dehydrogenase module is quite similar to the mitochondrial Complex I (NADH:UQ oxidoreductase), and uses flavin mononucleotide (FMN) as its prosthetic group. Electron transfer from the hydrogenase module to the redox module is accomplished via a series of Fe–S clusters. The catalytic mechanism of SH has not been studied, but in Complex I (which operates in the opposite direction to SH, oxidizing NADH and reducing UQ) it involves intact hydride transfer from NADH to FMN. The reaction of SH in the forward direction presumably accumulates two electrons and two protons on FMN, followed by hydride transfer from reduced FMNH_2_ to NAD^+^. Thus in *C. necator*, water – not H_2_ – would be the source of hydrogen to FMNH_2_ and NADH.

In both photosynthesis and hydrogenase-based chemoautotrophy, then, NAD(P)^+^ is reduced by electrons transferred from iron–sulfur carriers to a flavin prosthetic group, with a hydrogen nucleus (H^+^ or D^+^) acquired from solution, and then by hydride transfer from the reduced flavin to the nucleotide in a fast catalytic step. We do not yet know whether the observed fractionations are the result of equilibrium or KIEs, either during reduction of the flavin ring or during hydride transfer to the nucleotide. Regardless, the fact that these two enzymes with similar prosthetic groups express similar fractionations is arguably not a coincidence. Given the apparent constancy of the fractionation across a wide variety of plants, photosynthetic algae and bacteria, and (now) chemoautotrophic bacteria, we suggest that it may represent a general feature of many enzymes that use flavins as redox carriers.

## Conclusion

*C. necator* H16 growing aerobically on H_2_ + CO_2_ exhibited net D/H fractionations between fatty acids and water that range from -199‰ to -236‰ (average -217‰), indistinguishable from those observed in most photoautotrophic algae and bacteria. Diverse lines of evidence, including examination of known biochemistry, study of hydrogenase mutants, and fractionation curves developed by modulating the isotopic composition of growth water, all indicate that H_2_ contributed little or no hydrogen to lipids. Instead, the observed fractionation must have resulted from the reduction of NADH with hydrogen nuclei from water, using only electrons supplied by H_2_. Small differences in fatty acid δD values (≤25‰) were observed between hydrogenase-deficient mutants and the wild type. These differences can likely be explained by changes in growth rate or efficiency, cellular localization of H_2_ oxidation, and/or competing demands for NADH. The lipid–water fractionations exhibited by *C. necator* are common, though not ubiquitous, among chemoautotrophs for which fractionations have been reported. The hypothesis that chemoautotrophic and photoautotrophic biomass can be distinguished by their δD values is therefore falsified, although at least some chemoautotrophs are significantly D-depleted for reasons not yet known. A fractionation of roughly -200‰ appears to be common to many NAD(P)^+^ reductases.

## Author Contributions

BC planned the experiments, conducted research, analyzed the data, and wrote the manuscript. AS performed lipid analyses, analyzed the data, and wrote the manuscript. DF helped with microbial cultures and gas isotope analyses. BP provided analysis of genomic data for strain H16. QQ cultivated and sampled microbial cultures. MK performed the polar lipid analysis, DV planned and supervised the experiments, analyzed the data, and edited the manuscript.

## Conflict of Interest Statement

The authors declare that the research was conducted in the absence of any commercial or financial relationships that could be construed as a potential conflict of interest.

## Supplementary Material

**FIGURE S1 |** Bar charts of hydrogen-isotopic fractionations between fatty acids and growth water, grouped by fatty acid structure and color-coded by strain. Top, middle, and bottom plots are data from cultures with no D-enrichment, medium D-enrichment, and high D-enrichment, respectively.

**TABLE S1 |** Complete dataset of fatty acid structure, abundance, and isotopic composition; polar lipid structure and abundance; and bacterial growth measurements.
