# Supplementary material for: Minimal Influence of [NiFe] Hydrogenase on Hydrogen Isotope Fractionation in H2-Oxidizing Cupriavidus necator
Source: Front Microbiol. 2017 Oct 4;8:1886. doi: 10.3389/fmicb.2017.01886 (PMC5649130; doi:10.3389/fmicb.2017.01886)

Supplementary Figure S1. Bar charts of hydrogen-isotopic fractionations between fatty acids and growth water, grouped by fatty acid structure and color-coded by strain. Top, middle, and bottom plots are data from cultures with no D-enrichment, medium D-enrichment, and high D-enrichment, respectively.

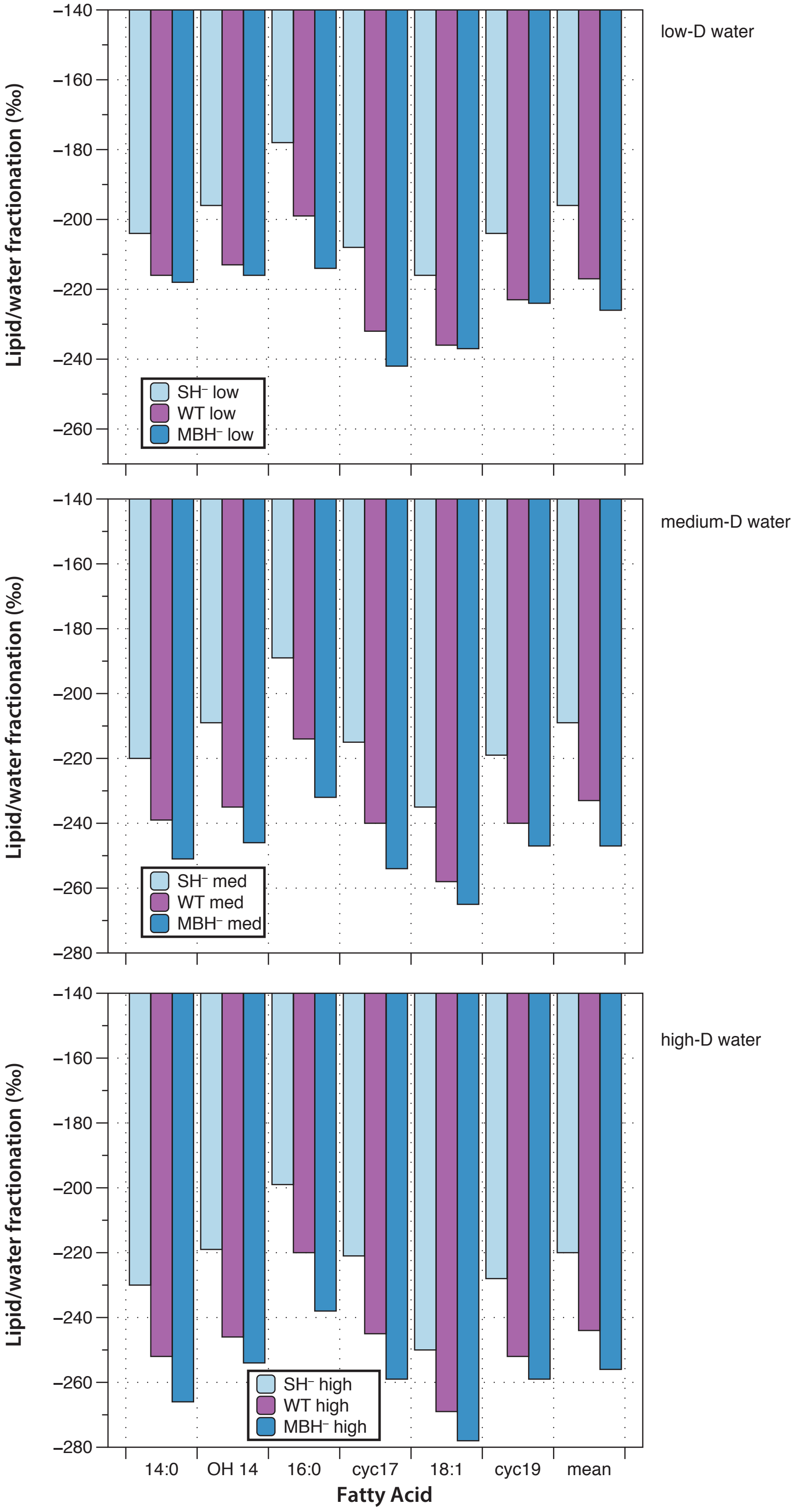

Supplement: Supplementary file 1 [file Image_1.pdf]
